# *“Everything else is going to be ok if your spiritual wellness is well”*. A qualitative exploration of wellness amongst secondary school students in Fiji

**DOI:** 10.1080/17482631.2021.2001895

**Published:** 2021-11-21

**Authors:** Latileta Odrovakavula, Masoud Mohammadnezhad

**Affiliations:** a Public Health, School of Public Health and Primary Care, Fiji National University, Suva Fiji Islands; b Public Health (Health Promotion), School of Public Health and Primary Care, Fiji National University, Suva Fiji Islands

**Keywords:** Adolescents wellness, wellness dimension, ideal wellness programme, Fiji

## Abstract

**Purpose:**

Adolescent wellness over the years has been a major public health concern. The adolescent period is stated to be a critical phase as developments occur in all areas of the individual. This study aims to explore secondary school students’ perceptions on knowledge of wellness, influences of wellness, prioritization of wellness dimensions and ideal adolescent wellness programmes in Fiji.

**Method:**

This study qualitatively investigates adolescents in four purposively selected schools in Suva, Fiji. 31 students who enrolled into years 11 to 13 in the selected schools were interviewed in-depthly using a semi-structured, open-ended questionnaire. Data was transcribed and classified into categories, sub-themes and major themes.

**Results:**

Participants’ responses indicated wellness perceived as multidimensional with no regard to dimensional balance. Family, peers and school support, social media, nutrition and physical activities are factors perceived to influence wellness. The availability of school counsellors, facilitation of effective school health programmes, and incorporation of wellness into the school curriculum were perceived as ideal ways to increase adolescent wellness.

**Conclusions:**

This study provides important research findings of adolescent wellness for Fiji’s health sector, its policymakers and programme developers. Further studies are needed to understand the broad concept of wellness and its several dimensions.

## Introduction

Wellness is defined as viewing individuals and their health from a holistic perspective that includes several dimensions involving the mind, body, spirit and community interactions or social bonds (Bezner, 2015; Kirsten et al., 2009; Roscoe, 2009). Globally, the wellness concept is being integrated into health systems with the holistic approach now considered the preferred approach to address health issues, transforming the notion of what constitutes health, its concepts and services (Anspaugh et al., 2004; Baldwin et al., 2017). Adolescent wellness is increasingly seen as a major public health concern (WHO, 2018). The adolescent period is stated to be a critical phase as developments occur in all areas of the individual (Pinhas-Hamiel & Zeitler, 2005). Coupled with these developments are changes in health and health-related behaviours that influence adult onset of diseases and poor health (Chilton et al., 2014; Patton et al., 2016). These include uptake of health-risky behaviours such as smoking, drug use, poor diet and exercise and unsafe sexual practices (El Achhab et al., 2016; Rosario et al., 2014; Salam et al., 2016).

It is also vital to understand adolescent perceptions of wellness (Spurr et al., 2012). Based on changing health behaviour theories, the literature states, an individual’s perceptions influence health behaviour (Ferre & Klein, 2015). This same principle has been found to influence an individual’s wellness status. Perceived health is a predictor of morbidity and mortality, and wellbeing (Tennebø et al., 2013). Adolescent perception of health can serve as evidence that informs policies and programmes (Ott et al., 2011). For Fiji, the Ministry of Health and Medical Services (MoHMS) has developed adolescent health through public and private efforts such as the Fiji Adolescent Health Programme. Review of these programmes, indicated that adolescent health is largely focused on reproductive health and most do not address the broader adolescent health needs of communicable and chronic diseases and mental health (Ministry of Health and Medical Services, 2016).

However, there is currently limited research focusing on adolescent wellness in Fiji. The research gap remains for wellness, globally and in Pacific Island countries and Fiji. There is abundant literature available on the factors that influence wellness dimensions, however, there are limited studies available relating to overall wellness and the various dimensions. This paper aims to explore perceptions on wellness amongst secondary school students in Fiji.

## Materials and methods

### Study population

Adolescents (n = 31) from four secondary schools in Suva and the greater Suva area participated in this study. The greater Suva area includes Suva, Lami, Nasinu and Nausori (Fiji Roads Authority, 2015). To select the schools, multi-cluster sampling was used. The first cluster considered the geographical location of the schools and these were divided into the four areas of Suva, Lami, Nasinu and Nausori. The second cluster considered the urban or peri-urban locations of the schools. A list from the Ministry of Education, Heritage and Arts (MEHA) detailed the locations of the schools. From this list, study sites were purposively selected to ensure there were representatives of both peri-urban and urban schools. It was also ensured that there was representation of ethnic, religious and gender groups.

Students who were willing to participate were purposively sampled provided they were of Fijian nationality and enrolled into years 11 to 13 in the selected schools. Participants in the higher age category and those enrolled in the higher class levels have been observed to make sense of their development when compared to younger ones (Hockenberry & Wilson, 2007). Sampling was conducted regardless of gender, religion or ethnicity. Sampling was conducted until data saturation was achieved (Aldiabat, 2018).

### Study procedures

Upon ethical approval, endorsement was sort from MEHA research and development section. Courtesy visits to the study sites allowed for arrangements for data collections. Students were introduced and informed about the study verbally and through information sheets which explained the purpose and study importance. Consent forms to obtain parent or guardian approval were provided to students below 18 years old. These consent forms were printed in three languages: English, *iTaukei* and Hindi. A semi-structured, open-ended questionnaire was used to guide interview questions. The use of open-ended questions allowed the facilitator to add or skip questions depending on the answers received from the participants (Reja et al., 2003). The questions were developed to evaluate and assess the perceptions of adolescent wellness amongst students based on a previous study conducted on adolescent wellness (Spur, 2009). There was a total of six sections and 11 questions, these are presented in Table I. The tool was in the English language. The in-depth interview was conducted in a conducive environment that was non-threatening and away from any third-party intrusion. The researcher conducted the interview with interview times varying from 20 to 30 minutes.

**Table I.** In-depth interview questions

Sections	Questions
Perception of Wellness	What does Wellness mean to you? What are some Barriers /supportive factors/ recommendation to your wellness?
Physical Wellness	What do you understand about the terms physical Wellness? What factors do you think can positively or negatively influence your physical wellness?
Psychological Wellness	What do you understand about the terms psychological wellness? What factors do you think can positively or negatively influence your psychological wellness?
Social Wellness	What do you understand about the term social wellness? What factors do you think can positively or negatively influence your social wellness?
Spiritual Wellness	What do you understand about the terms spiritual wellness? What factors do you think can positively or negatively influence your spiritual wellness?
Overarching Question	In terms of the dimension of wellness that we have discussed, which one do you think is important or is priority?

### Data management and analysis

Data recorded, using voice recorders and hand-written notes, were analysed. These audio recordings were transcribed and thematic analysis applied. Several meetings were held between the principal researcher and research supervisor to discuss findings and to determine taxonomy of perceptions. Coded data and data matrices were used to explore patterns of wellness perceptions. Data was classified into categories, sub-themes and major themes (Nowell et al., 2017).

### Ethical consideration

Consent forms to obtain parent or guardian approval were provided to students, below the age 18 years. These students were also provided assent forms to indicate their willingness to participate in the study. Participants’ names and their responses were kept confidential.

## Results

### Characteristics of the participants

Thirty-one students participated in the face-to-face interview. There was an equal number of participants from each school with an average of eight student participants per school. Mean age of participants was 17 years with more female participants (74.2%) compared to male student participants (25.8%). The majority of the participants were enrolled into years 12 (35.5%) and 13 (64.5%). Students were either Fijian of *iTaukei* descent (64.5%) or Fijian of Indian descent (35.5) (Table I). To protect confidentiality, each participant was assigned a number from Participant 1 to Participant 31 and presented in this study as P1, P2, P2 … The year in which the students were enrolled is also indicated in the quotation references. (Table II)

**Table II.** Characteristics of student participants (n = 31)

Characteristics		Frequency	Percentage
Schools	*School 1 (Participants 1–7)*	7	22.6
	*School 2 (Participants 8–15)*	8	25.8
	*School 3 (Participants 16–23)*	8	25.8
	*School 4 (Participants 24–31)*	8	25.8
Gender	*Male*	8	25.8
	*Female*	23	74.2
Age	*17 years old*	16	51.6
	*18 years old*	12	38.7
	*19 years old*	3	9.7
Ethnicity	*iTaukei*	20	64.5
	*Indo-Fijian*	11	35.5
Education Level	*Years 12*	11	35.5
	*Years 13*	20	64.5

### Themes identified

The final analysis revealed three dominant themes areas: knowledge, prioritization and influence of wellness dimensions. Table III summarizes these themes, their related sub-themes and categories.

**Table III.** Themes, sub-themes and categories of in-depth interview analysis

Themes	Sub-themes	Categories
Knowledge of wellness	Concept of wellness	The multi-dimensions of wellness
		Physical fitness as physical wellness
		Right mindset essential for psychological wellness
		Good relationships central for social wellness
		Spiritual wellness is associated with a higher power
	Characteristics of wellness	Being physically fit
		Balanced diet
		No substance use
		Resilient spirit
		Sharing problems
Influences on wellness	Influence of physical wellness	Physical activity as core
		Poor diet and diseases
		Glue sniffing and marijuana as common drug
		Excessive use of social media
	Influence of psychological wellness	Lack of family support
		Peers and social issues
		Self-concept
		Stress leading to burnout and suicide ideation
	Influence of social wellness	Receiving family support
		Teachers as counsellors
		Social media expectations
	Influence on spiritual wellness	Family teachings
		Negative impacts from peers and friends
		Social media exposure
Prioritization of wellness dimension	Spiritual wellness as priority	Resilient spirit for right decisions
		Builds character
		Relationship with God as important

## Theme 1: Knowledge of wellness

### Concepts of wellness

Wellness definitions provided by the participants were not limited to physical fitness. In describing wellness, students included mental, physical and social health. Students also perceived wellness to include protection from harm existing in one’s environment and essential for decision-making.

Wellness sort of means the state of physically, mentally, spiritually [sic] wellbeing. Like you are able to think out what you’re doing, your actions, you know what you are doing. You know what you are taking in, the capability of planning for the future and also for today. (P 28, Year 13 student)

Wellness was commonly defined as being physically fit. The majority (n = 24/31) of the participants viewed wellness as how one maintained physical health. Participants stated that it was important to physically maintain one’s body through exercise. *Physical wellness* was understood to be the ability to do any form of physical activity such as walking. The majority of participants (28/31) stated that physical wellness, involved physical fitness. Physical wellness was perceived to be about the human body and how capable it is of carrying out physical activities:

Things that you do physically, you are able to walk, and do anything. (P 1, Year 13 student)

Students stated that *psychological wellness* reflected thoughts and the controlling of thoughts, this in turn influenced decision-making. Apart from mental health, students perceived that psychological wellness influenced other areas of a person’s life. Not being psychologically well was associated with behaviour such as drinking.

Being in the right mind. Psychological wellness leads to spiritual, physical, and other areas because you have to be in the right mind. If I wasn’t psychologically well, I’d probably be drinking and all that. I wouldn’t probably believe in God. (P 21, Year 12 student)

*Social wellness* was understood by the relationships one has with friends. It is the support provided to an individual through peers, parents and teachers. Having good relationships was indicated to be central for social wellness, this was in terms of family, friends and community.

There is equal, like there is no damages in your family. You have good relationships with your family. (P 11, Year 13 student)

*Spiritual wellness* was understood by most of the participants as a relationship between humans and God. A Year 12 student talked about *“Your connectivity with a higher, deeper power”* as spiritual wellness. However, the understanding of spiritual wellness was not limited to religion. The term has also been defined as dealing with one’s own personal values.

Spiritual wellness is wellness within yourself, your soul, maintaining, how you are in decision-making, the parts in decisions where you can decide what’s right and wrong. (P 9, Year 13 student)

### Characteristics of a well person

The majority of the participants (n = 18/31), identified physical activity, being physically fit and eating a balanced diet as characteristics of a well person. Four participants identified that a well person would care for their psychological health. This was seen to be through the avoidance of drug as they are perceived to affect the mind and decision-making.

Not taking drugs that can affect your mind, so that you can make good decisions in life and social wellness, having balance or proper conversations with your family.” (P 11, Year 13 student)

Three students stated that a well person has a resilient spirit when faced with challenges. Students talked about the ability *“to bounce back”*. Having a resilient spirit was defined as being:

Fit in all aspects of your life. You are fit, mentally, socially, spiritually. You having a strong spirit. (P 12, Year 13 student)

Sharing social problems with others was also observed to be characteristics of a well person.

Talking to someone about your problems … (P 17, Year 12 student)

## Theme 2: Influence on wellness dimensions

### Influences of physical wellness

Students’ responses can be categorized into four categories: influences of physical activity; nutrition; drugs and substances; and social media. The types of physical activities thought as beneficial to physical wellness were not limited to physical fitness. Students identified fitness regimes such as attending gyms on a regular basis, playing sports and doing chores as forms of physical activities. Participants stated that any activity could be carried out to be physically active.

The school environment was perceived to be supportive towards physical wellness as the school curriculum promotes activities such as physical education classes. One school has also offered their students physical activity programmes as an extracurricular activity.

We had the taekwondo programme, it was done here after school, 3[pm] to 5[pm]. You pay $1.50 for two hours. It’s a self-defence programme. (P 8, Year 12 student)

Participants identified snacks and junk food to be harmful to health. Poor Nutrition has also been stated to influence the onset of diseases on the human body. One student stated:

Eating the wrong type of food that will lead them to NCD, even children nowadays have diabetes, so if they are well, they wouldn’t have these types of sickness. (P 30, Year 13 student)

Students perceived substance use such as drugs, glue-sniffing and smoking marijuana as influencing physical wellness. These have been identified as common substances among high school students.

For substance use, we don’t face that as a problem here in school, but we hear it is common in other schools, we hear cases of students sniffing glue, because that is the common one and could be marijuana because they can access to this thing easily. (P 29, Year 13 student)

In addition, physical wellness was perceived to be impacted by extensive use of technology and gadgets. Participants stated that students spend too much time using technology and gadgets, influencing their physical wellness. Students also perceived that there were increasingly attracted to social media today. More time is spent online leading adolescents to sit for long hours and reducing their chances of physical activity.

### Influences of psychological wellness

Not being supported in making career choices and the high number of home responsibilities have been stated to influence adolescent psychological wellness. One student (P 24, Year 12 student) stated:

My mother is sick; she is in the hospital. We are three brothers. I’m impacted because we do the housework and at the same time we study. Now my mother’s foot has been cut because of diabetes and now for the rest of her life we’d be doing the work, just have to cope, motivation from the teachers.

In addition, issues such as divorce and poor parental behaviour were stated to have negative influences on one’s mental health.

The first time I heard that my dad did something wrong, and I was really angry. It affected my mind and my life, my aunt told me, just to believe in my dad as it was a mistake. (P 4, Year 13 student)

Apart from family, participants also talked about having friends who encouraged risky behaviours that contradict parental teachings. Participants perceived being involved with the wrong crowd caused social issues, leading to internal conflict.

Support from home and being in the right friends’ groups. Your parents can be in the right, but your friends can say that what they say is wrong and that can lead to conflict with your family and the mind doesn’t know whether it’s this or that but because it’s friends, friends usually come before family. (P 21, Year 12 student)

Participants also stated that having friends to share life experiences was beneficial for one’s psychological wellness. Students talk to their friends as a coping mechanism for common issues.

At home, my parents don’t really pay attention to me, and what people say about me. My friends, we face the same things, and we just all talk about it. Maybe if someone can talk to my parents to understand what a teenage girl goes through now instead of in the past. (P 23, Year 12 student)

There is also recognition that one’s own choice, student’s own decision-making, influences their psychological wellness. Participants stated that self-concept is important as it allowed people to handle challenges, which in turn influenced decisions.

You, yourself because it’s your mentality, it’s your job to know how like people say a lot of hurtful things, that can affect you, you should be strong and push that away … (P 17, Year 12 student)

The findings indicate that students recognized the impact of stress on their psychological wellness. Participants identified these impacts as including burnout and suicide ideation. This has been associated with academic work. One student stated:

Many at times, I’d lose hope in myself that life is going to end because of stress. But then I see my parents as my priority and then you know, this is not right, I have to do it. Many at times I lose my mental health, like this exam is hard but then I have to do it, because I have to be in a position to support my family, someone good, I have to set an example for others and my parents will be proud. (P 27, Year 13 student)

### Influences of social wellness

Apart from peer support, students perceived support from their families as influencing their social wellness. For participants who perceived that obtaining social wellness support at home was feasible, they spoke about receiving care and attention and being advised on life choices and behaviours.

My father is a pastor, so he is my counsellor, I can also tell my friends what he tells me. (P 22, Year 12 student)

Expectations on students, whether it be academic or home responsibilities, were barriers towards their social wellness. Students provided statements such as:

For all teenagers, it’s the students that is expected to study so much but is believed to have no personal duties at home and then at home, when you try and study, you got duties to do and then you have parents that tell you that, you know when we were at your age, we did this. Then we try to struggle and balance, because maybe school was harder in the past but then it’s harder now too, because of technology advancing. (P 21, Year 12 student)

The lack of family support was stated to lead to social issues where one student stated:

For some they don’t get the love and care from their parents so they look for love and care from elsewhere. (P 29, Year 13 student)

Students perceived that social wellness is promoted when they receive support from their family which directs their daily lives and choices, although not all students feel supported.

Teachers’ support was perceived to be influential in participants’ decision-making and behaviour. Teachers have also been perceived to be school counsellors providing emotional support to students. However, participants stated there were only certain teachers whom they perceive as counsellors.

My school makes it easy for me to be well, my teachers are good, my friends are good, and I like this combination. They really support you. We don’t have school counsellors but we are open to our teachers. (P 12, Year 13 student)

Social media has also been stated to influence adolescent social wellness. Participants also felt the need to spend more time online as theirs peers.

Social network, even from my opinion, I spend 3 to 4 hours on social media, my parents tell me to quit it but my friends, you can’t leave friends behind. (P 27, Year 13 student)

### Influences of spiritual wellness

Participants have stated that the family environment and parental guidance as means of spiritual wellness development. One student stated spiritual wellness began at home as family is structured to raise children to be spiritual.

For us Muslims we need to pray five times a day and follow all Muslim practices, my parents encourage us. (P 30, Year 13 student)

Several adolescents expressed the negative influence of peers on spiritual wellness. Time spent with the “wrong crowd” were stated to have negative impacts on spiritual wellness.

By following the wrong crowd, instead of going to church you go to the store/shop. (P 3, Year 13 student)

There was recognition made towards joining youth groups and attending youth programmes, these were stated to positively influence one’s spiritual wellness.

Going for junior MYF [Methodist Youth Fellowship] and Bible studies can help you with your spiritual wellness. (P 22, Year 12 student)

Social media has been viewed to have a negative impact on spiritual wellness. Adolescents felt that spending excessive time on social media increased their exposure to various content.

## Theme 3: Prioritization of wellness dimension

### Spiritual wellness as priority

Around half of the participants prioritized spiritual wellness. Students believed that achieving spiritual wellness would influence other dimensions. Spiritual wellness was perceived as developing a resilient spirit; this was seen to be essential as it influenced decision-making.

Spiritual, because the stronger your spirit is, the more you are able to make the right decisions. Everything else is going to be ok if your spiritual wellness is well. (P 12, Year 13 Student)

Spiritual wellness was also perceived to influence students’ life at home and at school. Religion-affiliated spiritual wellness also influenced students to prioritize spiritual wellness.

Spiritual it helps you become a good daughter at home, school students and a good person overall (P 31, Year 13 student)

## Discussion

This study aimed to explore adolescent perceptions of wellness. The results highlighted three main themes: knowledge of wellness, prioritization of wellness and influence of wellness.

### Knowledge and prioritization of wellness

This study showed that wellness was perceived to be multidimensional, however the definitions were limited to a mixture of two or three dimensions, with no discussion relating to all seven dimensions defined in the Fiji MoHMS wellness framework (Ministry of Health and Medical Services, 2015). The predominant definition was wellness as physical health, where students were able to associate diet, exercise, physical fitness and substance use as factors influencing wellness. This may potentially explain why physical wellness characteristics were mostly associated with overall wellness. This is similar to other studies where findings indicated physical wellness as the most common dimension (Brett, Horton & Snyder, 2009; Preskitt et al., 2015).

In this study, wellness as a concept was not perceived as a balance of dimensions but the existence of good health. Students picking a dimension superior to another supported this. Although physical wellness was the predominant wellness definition, students felt spiritual wellness was the most important dimension. Participants identified with spiritual wellness at a personal level as the dimension meant a closer proximity to God, building character and influencing individual decisions. Findings indicated the need to empower students to perceive that for wellness to occur there needs to be balance between all dimensions (Ministry of Health and Medical Services, 2014).

### Influence of wellness

The results of this study found that physical activity was perceived to influence physical wellness as supported by literature (Andrade et al., 2010; Miller & Foster, 2010). Perceptions of physical activities were not limited to exercise; this aligns with Fiji’s MoHMS advocacy messages on physical health, which states that physical activity could be done anywhere, with no requirement of clothing or equipment (Ministry of Health and Medical Services, 2014). In terms of nutrition, the results of this study found associations made between eating the wrong types of food and NCDs. NCDs are a major concern for Fiji and the Pacific with the region declared as experiencing an NCD crisis in 2011 (Tolley et al., 2016). It is essential that adolescents recognize risk factors associated with NCDs. Findings of this study indicated the need to review food sold in school canteens. These includes the review of food and nutritional policies such as the Fiji School Health Policy(2016) and the School Canteen Guideline(2013). In addition to nutritional influences, the results of this study found social media to influence adolescent physical wellness. The adolescent age group make up 77% of Fiji’s social media users (Tarai, 2015). High school students who spend more time on social media are less likely to participate in physical activity as compared to those who are non-social media frequent users (Shimoga et al., 2019). This study’s results also highlight the perception of substance use influencing physical wellness. Students felt that although they may not be observing drugs and substance use occurring within their school environment, they are aware that their peers in other schools are engaging in such activities. This finding may potentially indicate the high use of drugs as found by the 2016 Global School-based Student Health Survey conducted in Fiji, which identified that nearly half of high school students had consumed drugs (57%) and alcohol (49%) before the age of 14 (WHO, 2016).

In terms of psychological wellness, the outcomes of this study indicated that high number and sudden change of home responsibilities and the lack of family support affect students mentally. These findings indicate the need to address family issues and the need to increasing family functionality (Shek, 2002). Previous research carried out in Fiji observed there are pressures on Fijian young people to perform well in school, as well as continually be there as support for family and peers and sometimes the pressure to do both is overwhelming (Grambeau, 2007). This has led to depression, anxiety and suicide ideation (Nooney, 2005; Polanco-Roman et al., 2019; Thapar et al., 2012). These findings suggest more needs to be done to enable adolescents to manage and control stress. Participants have indicated sharing problems with the peers was a coping mechanism where students have the opportunity to share life experiences amongst same age groups. Peers were perceived to have a better understanding of adolescent issues (Bakalım & Taşdelen-Karçkay, 2016; Chow et al., 2013). Adolescent wellness programmes are to consider peer support interventions.

In terms of social wellness, this study’s result found that adolescents receive social support from their peers, however, issues of peer pressure have been perceived to encourage negative behaviour such as smoking, drinking and drug use (Moore et al., 2018). Apart from peers, students felt receiving social support from their families would influence their social wellness. This highlights the importance of positive family relationships, where low levels of conflict, high levels of support and open communication exist (Lorenz et al., 2006; Sacks et al., 2014; Thomas et al., 2017). Results of this study indicated that there are higher chances of looking for social support outside of the home. This finding suggests family support towards adolescents’ needs improvement (Bean et al., 2003).

The results of this study found that participants perceived family to play a major role in their spiritual wellness. Students perceived home upbringing being centred on spirituality, where one is taught religion and spirituality from a young age. Fiji, like its Pacific Island neighbours, has been stated to be a religious country (Newland, 2006). This may have influenced society and their approach towards child rearing (Horwath et al., 2008).

## Study limitations

The findings of this research must be interpreted within the context of its limitation. Due to the setting and the nature of the study participants, the timing for data collection was limited. Due the study being cross-sectional, it is limited to high school students and findings may not be generalized to the adolescent population in Fiji.

## Conclusion

Wellness was perceived to be more than physical exercise and the consumption of a balanced diet; wellness was perceived to be multidimensional. The predominant definition was wellness as physical health, where students were able to associate nutrition, exercise, physical fitness as factors influencing wellness. Psychological wellness was influenced by the high number and sudden change of home responsibilities, lack of family support and high expectations. Social wellness was influenced by support from family, friends and school. Spiritual wellness was influenced by family teachings, peers and social media. Physical wellness was perceived to be influenced by physical activities, nutrition, substance use and social media.

This study adds to literature as it provides important research findings on adolescent wellness to Fiji’s health sector, its policymakers and programme developers, however, research gaps remain. Further studies are needed to understand the broad concept of wellness and its several dimensions. The knowledge level, the perceptions and practice of wellness amongst other population groups remain unanswered.
